# HIV and/or AIDS-related deaths and modifiable risk factors: A descriptive study of medical admissions at Oshakati Intermediate Hospital in Northern Namibia

**DOI:** 10.4102/phcfm.v7i1.883

**Published:** 2015-09-25

**Authors:** N.K. Mgori, Robert Mash

**Affiliations:** 1 Division of Family Medicine and Primary Care, Stellenbosch University, South Africa; 2 Oshakati Intermediate Hospital, Namibia

## Abstract

**Background:**

High rates of HIV infection have decreased life expectancy in many African countries. Regardless of worldwide efforts to escalate treatment, care and prevention strategies, the number of deaths due to AIDS-related disorders is still high. Local healthcare workers suspect that there are modifiable factors in the care of HIV and/or AIDS patients which can be identified and improved.

**Aim:**

To describe the HIV and/or AIDS-related causes of adult mortality and identify modifiable factors amongst patients admitted to Oshakati Intermediate Hospital, northern Namibia.

**Methods:**

Data was extracted retrospectively and coded using the modified CoDe protocol for AIDS. Modifiable factors relating to the patient, health system or clinical care were identified using a standardised data collection tool.

**Results:**

A total of 177 HIV and/or AIDS patients were identified, 94 (53.1%) were male and 120 (68%) had a CD4 count of less than 200 cells/mL. The common HIV-related causes of death were tuberculosis (25.9%), renal failure (15.8%), *Pneumocystis jirovecii* pneumonia (11.3%), cryptococcal meningitis (9%), HIV wasting syndrome (7.9%) and AIDS-defining malignancy (7.9%). The analysis revealed 281 modifiable factors; patient-related factors were the most common (153 [54.4%]), followed by health system factors (97 [34.5%]) and healthcare personnel factors (31 [11%]).

**Conclusion:**

Our findings have highlighted the challenges in overall HIV and/or AIDS inpatient care and surrounding primary care facilities. The identification of specific modifiable factors can be used to reduce mortality by providing training as well as rational monitoring, planning and resource allocation.

## Abstract (fr)

**Décès liés au VIH/SIDA et facteurs de risques modifiables: Etude descriptive des admissions médicales à l’Hôpital intermédiaire d’Oshakati au nord de la Namibie.**

**Contexte:**

Le taux élevé d’infection par VIH a réduit l’espérance de vie dans de nombreux pays africains. Malgré les efforts mondiaux d’intensification des traitements, des soins et des stratégies de prévention, le nombre de décès dus aux troubles associés au SIDA est encore élevé. Le personnel de santé local pense qu’on peut identifier et améliorer certains facteurs modifiables dans les soins des malades du VIH/SIDA.

**Objectif:**

Décrire les causes de mortalité des adultes liées au VIH/SIDA et identifier les facteurs modifiables chez les patients admis à l’Hôpital Intermédiaire d’Oshakati, au nord de la Namibie.

**Méthodes:**

les données ont été extraites et classées rétrospectivement en utilisant le protocole modifié CoDe pour le SIDA. Les facteurs modifiables concernant le patient, le système de santé ou de soins cliniques ont été identifiés à l’aide d’un outil de collecte de données standardisé.

**Résultats:**

On a identifié au total 177 malades du VIH/SIDA, dont 94 (53.1%) étaient des hommes et 120 (68%) avaient un taux de CD4 de moins de 200 cellules /ml. Les causes les plus fréquentes de décès lié au HIV étaient la tuberculose (25.9%), l’insuffisance rénale (15.8%), la pneumonie *Pneumocystis jirovecii* (11.3%), la méningite cryptococcique (9%), le syndrome de dépérissement lié au VIH (7.9%) et les tumeurs liées au SIDA (7.9%). L’analyse a révélé 281 facteurs modifiables; les facteurs liés au patient étaient les plus fréquents (153 [54.4%]), suivis des facteurs liés au système de santé (97 [34.5%]) et ceux liés au personnel de santé (31 [11%]).

**Conclusion:**

Nos résultats ont mis en évidence les défis des soins en hospitalisation des malades du VIH/SIDA et dans les établissements de soins primaires avoisinants. On peut utiliser l’identification de facteurs modifiables particuliers pour réduire la mortalité en offrant des formations, des contrôles rationnels ainsi que la planification et l’allocation des ressources.

## Introduction

HIV and/or AIDS is one of the major global public health problems and causes substantial morbidity, mortality, negative socio-economic impact, and human suffering.^1,2^ Since the beginning of 1981, HIV and/or AIDS has become a pandemic disease, claiming some 20 million lives.^1^ By the end of 2010, an estimated 34 million people of all age groups (31.6 million–35.2 million) were living with HIV worldwide.^1,2^

Sub-Saharan Africa, with only 12% of the global population, ranks top amongst the regions that are drastically affected by HIV.^3^ The region contributes 68% of all HIV and/or AIDS patients in the world, whilst South-East Asia has 12%, Eastern Europe and Central Asia 4%, Latin America 4% and North America 4%. South Africa has the highest number of people living with HIV and/or AIDS (having more than 4 million patients).^2^

Namibia is amongst the countries which are significantly affected by HIV and/or AIDS. It has a relatively small population of 2.1 million and the Ministry of Health and Social Services estimates the HIV prevalence rate to be 18.2%.^4^ Furthermore, in 2011–2012 it was estimated that 193 000 adults and children were living with HIV. This number is predicted to increase to 196 000 in the year 2015–2016.^5^

The HIV and/or AIDS epidemic is the largest contributor to the burden of disease in Namibia, contributing 37% to indirect causes of maternal mortality, which is an increase of 50% from 2000 to 2006. Furthermore, life expectancy has decreased from 62 years in 1996 to 44 years in 2006 due to HIV and/or AIDS.^4,5^

Over the past two decades, the government of Namibia has prioritised HIV and AIDS in its development goals for 2030. This has led to increases in universal access to antiretroviral treatment, with coverage of between 50% and 80% of eligible patients.^2^

Strategies used to curb HIV and/or AIDS morbidity and mortality in Namibia include the provision of free, highly active antiretroviral therapy (HAART); recruitment, re-training and retention of health workers; health education; screening for and ultimately treating opportunistic infections as well as sexually transmitted diseases.^3,4^ The screening for opportunistic infections and preventive therapy is offered by both hospitals and clinics and includes the provision of isoniazid, co-trimoxazole, fluconazole and malaria prophylaxis in pregnancy. Ongoing adherence counselling and the retention of patients at the clinic level may also help to reduce mortality and morbidity.^4,5,6,7,8,9,10,11,12,13^

Despite the national efforts, premature deaths from HIV and/or AIDS continue and reflect deficiencies in quality of care at different levels of the health system.^4^ Quality improvement projects have directed their energy at the clinic level, where most of the HIV clients are clinically stable, whilst less effort has been directed at those admitted to hospital with acute conditions.^5^

Like any other chronic condition, the management and progression of HIV and/or AIDS has both non-modifiable and modifiable factors associated with it. Modifiable factors are omissions or commissions that occur during the process of patient care and which subsequently lead to substandard care. They could be related to the patient, their caregiver, healthcare workers or the health system.^6,13^

Non-modifiable risk factors include age, race and gender. Modifiable non-HIV specific factors include smoking status, body mass index, hypertension, diabetes mellitus, and hepatitis C and B status. Other modifiable HIV-specific factors include, for example, the patient’s CD4 count and viral load.^14^

There has been no formal study to assess the causes of death and modifiable factors related to inpatient mortality amongst HIV-infected patients in Namibia. Local healthcare workers suspect that there are many modifiable factors affecting patient outcomes within Oshakati Intermediate Hospital. Identification of these modifiable risk factors and prompt interventions may provide insight into how to improve the management of HIV and/or AIDS patients admitted in our hospital.^14,15^

The aim of the study, therefore, was to investigate the modifiable factors contributing to HIV and/or AIDS-related deaths amongst adults in Oshakati Intermediate Hospital.

## Methods

### Study design

This was a descriptive study using data extracted retrospectively from the medical records of patients who died of HIV-related disease.

### Study setting

The study was undertaken at Oshakati Intermediate Hospital, which is located in Oshana region, northern Namibia. It is a 750-bed public hospital, serving approximately 750 000 Namibians and an unknown number of Angolans. The region has no district hospital; therefore, Oshakati Intermediate Hospital receives referrals from local primary care clinics as well as district hospitals in the Omusati, Kunene, Ohangwena and Oshikoto regions.

Oshakati Intermediate Hospital is also a teaching hospital; and has relatively more staff than nearby district hospitals. The internal medicine department alone has four specialist physicians, an HIV and/or AIDS clinical mentor, two medical practitioners, two interns and 14 nurses. The male and female wards have 130 beds with three high-dependency units. The HAART centre, oncology clinic and medical outpatient department are under the umbrella of the internal medicine department.

### Data collection

Folders from patients who had died of HIV-related causes were selected for data collection and only patients with a confirmed HIV-positive test result were included.

An auditing team was established that consisted of the researcher, four specialist physicians, an HIV and/or AIDS clinical mentor, an intern doctor, registered nurses and one administrator. This team was responsible for reviewing patient files from medical records and determining the diagnoses, HIV-related death and likely modifiable factors.

Basic clinical and demographic information was extracted for each patient in a standardised way: age, gender, HIV status, HAART status, length of stay in the ward, CD4 count and viral load.

The diagnoses were determined after a review of the patients’ medical history from the patients’ health booklets, nurses’ notes, clinical notes made by the admitting doctors, and progress reports from nurses and doctors at different points of care such as casualty, medical outpatients, inpatients and referral letters. The results of investigations, such as laboratory results and imaging, were reviewed. Results which were not in the files were traced manually using the available requisition numbers.

To date, no validation study exist to assess HIV-related causes of death in Namibia: The International Classification of Disease, ICD1-10, has limited value. Therefore, a protocol for determining the HIV-related cause of death, the CoDe system, was adapted from the Copenhagen HIV Programme, Centre of Global Excellence.^16^ This is the standardised system for classifying and coding deaths in studies of patients with HIV infection based on detailed data collection of information on the causes of death and contributing factors. The World Health Organization’s staging system was also used to categorise AIDS-specific causes of death.^2^ When there were disagreements between the reviewers, a consensus agreement with the clinical HIV mentor was reached. The mentor is an employee of the International Training and Education Center for Health in Namibia, covering the northern region.

The team then considered potential modifiable factors that could be related to the patients, their caregivers, their healthcare workers or the performance of the health system. The data collection tool and process used were modelled on similar approaches in the Child Healthcare Problem Identification Programme (CHPIP system), but adapted to adults with HIV.^15^ The study supervisor independently assessed the construction of the tool to improve its validity.

The causes of HIV and/or AIDS-related deaths were multi-factorial. As several factors might interact to contribute to deaths in this study, all modifiable factors were assessed per patient folder.

A pilot study was conducted to assess the quality of the clinical notes before the study. The study instrument was pre-tested a week before execution of the study on the records of patients with HIV who had recently died at the hospital. After reviewing 21 files for the pilot study, questions which were not clear were rephrased and retested; thereafter, a new questionnaire was constructed and used to capture the appropriate data for the study.

### Data analysis

All the data was captured on a purpose-designed form in Microsoft Access and this programme was subsequently used to produce a data spreadsheet in Microsoft Excel. The data was cleaned and verified to minimise entry errors, outliers and missing values. Frequencies and percentages were calculated for categorical data. Statisticians at the Centre for Statistical Consultation at Stellenbosch University were consulted for inferential data analysis. Inferential data analysis was done using the Statistical Package for Social Scientists software window version 19.0 (SPSS Inc., Chicago, IL). The frequencies and Pearson Chi square test were used to analyse the association between the dependent and independent variables and *p* < 0.05 was considered statistically significant.

## Ethical considerations

The proposal was approved by the Health Research Ethics Committee of Stellenbosch University (S12/11/301) and the Oshakati Intermediate Hospital ethics committee.

## Results

### General characteristics of HIV-infected patients

There were 2452 admissions, in both medical wards, during the period from 01 January to 31 December 2011. The overall number of deaths was 564 (23.0%), and of these deaths 201 (a case fatality rate of 35.7%) were documented as related to HIV. Out of these 201 deaths, the folders were retrieved for 177 patients.

Of 177 audited folders, 94 (53.1%) patients were on HAART and 48 (51.1%) had been initiated on HAART within the previous 90 days. Table 1 summarises the characteristics of the study population.

**TABLE 1:** General characteristics of the HIV-infected patients.

Characteristics	All (*n* = 177)	Males (*n* = 94)	Females (*n* = 83)
	*n* (%)	*n* (%)	*n* (%)
**Age group (years)**			
15–24	22 (12)	12 (13)	10 (12)
25–44	89 (50)	44 (47)	45 (54)
45–64	55 (31)	31 (33)	24 (29)
65	11 (6)	7 (7)	4 (5)
**Duration of hospital stay (days)**			
< 1	48 (27)	30 (62)	18 (37)
1–14	93 (52)	40 (43)	53 (57)
15–28	21 (12)	13( 62)	8 (38)
> 28	15 (8)	11 (73)	4 (27)
**CD4 counts (cells/mm^3^)**			
< 200	120 (68)	64 (53)	56 (47)
200 +	57 (32)	30 (53)	27 (47)
**HAART status**			
Pre-HAART	83 (47)	40 (48)	43 (52)
On HAART < 15 days	15 (16)	10 (67)	5 (33)
On HAART 15–90 days	33 (35)	15 (45)	18 (55)
On HAART 91–365 days	15 (16)	10 (67)	5 (33)
On HAART > 365 days	31 (33)	19 (61)	12 (39)
HAART (Total)	94 (53)	54 (57)	40 (43)

### HIV-related deaths

Amongst the patients reviewed, 82.0% died of HIV and/or AIDS-defining conditions, whereas 18.0% had non-AIDS-defining diseases. The underlying causes of death and frequencies are shown in Table 2. Extra-pulmonary tuberculosis accounted for most deaths, followed by renal failure, *Pneumocystis jirovecii* pneumonia, cryptococcal meningitis and HIV wasting syndrome.

**TABLE 2:** HIV-related deaths.

Diagnosis	*n* (%) (*n* = 177)
Extra-pulmonary tuberculosis	30 (16.9)
Renal failure	28 (15.8)
Pneumocystis pneumonia	20 (11.3)
Pulmonary tuberculosis	16 (9.0)
Cryptococcal meningitis	15 (8.5)
HIV wasting syndrome	14 (7.9)
AIDS-defining malignancies	14 (7.9)
Haematological disease	9 (5.1)
Chronic diarrhoea	8 (4.5)
CNS disease	7 (4.0)
Severe bacterial infections	5 (2.8)
Liver failure other than viral hepatitis	4 (2.3)
Alcohol abuse	3 (1.7)
Non-AIDS malignancy	2 (1.1)
Pulmonary thrombo-embolism	1 (0.6)
Cardiovascular disease	1 (0.6)

### Modifiable factors

Three broad modifiable factors were detected: patient- and caregiver-related factors accounted for 153 (54.4%), followed by healthcare system factors 97 (34.5%) and finally healthcare worker-related factors 31 (11.0%). Delayed presentation to a health facility was the commonest factor overall and accounted for 85.0% of patient- or caregiver-related factors. Failure to provide standard critical care services, haemodialysis or ventilator support accounted for 86.0% of health system factors whereas challenges experienced in monitoring patients was the main factor for healthcare workers (29.0%). Table 3 summarises the specific modifiable factors.

**TABLE 3:** Modifiable factors.

Modifiable factors	*n* (%)
**Patient- and caregiver-related factors (*N* = 153**)	
Delayed presentation to a health facility	130 (84.9)
Defaulted HIV treatment	21 (7.4)
Alcohol abuse	2 (1.3)
**Healthcare system factors** (*n* = 97)	
Lack of dialysis machine for HIV-positive patients	31 (31.9)
Inadequate respiratory support in the ward (oxygen supply)	26 (26.8)
Lack of ICU care for patient with HIV and/or AIDS	26 (26.8)
Inadequate blood product supply to the ward	6 (6.1)
Inadequate hospital stock of essential consumables	3 (3.1)
Basic laboratory investigations not available to ward 24 hours a day	2 (2.1)
Inadequate ward stock of essential consumables	1 (1.0)
Lack of standardised case management protocols in ward	1 (1.0)
Doctors in the ward inadequately supervised	1 (1.0)
**Healthcare worker factors** (*n* = 31)	
Essential treatment not prescribed	4 (12.9)
Inadequate monitoring of kidney disease	4 (12.9)
Inadequate monitoring/intervention of liver disease	3 (9.7)
Inadequate evaluation of treatable malignancy	1 (3.2)
Inadequate assessment of fitting or comatose patient	6 (19.3)
Inadequate fluid replacement	4 (12.9)
Insufficient case assessment and management in the ward	3 (9.7)
Inadequate switching HAART due to toxicity	2 (6.5)
Inadequate blood chemistry review	1 (3.2)
Doctor called, but did not respond	1 (3.2)
Doctor at peripheral hospital did not call referral hospital	1 (3.2)
Critical HIV patient not reviewed by doctor during weekend	1 (3.2)

Table 4 shows the relationship between variables and HIV clinical diagnosis. More male patients died of AIDS-defining diseases compared to females, and the difference was statistically significant, (57.24% versus 42.76%; *p*-value < 0.05), All age groups were at the same risk of dying either from AIDS-defining conditions or a non-AIDS-defining condition (*p* = 0.546). Moreover, for these two groups of patients there were no differences in length of hospitalisation (*p* = 0.99), In addition, more patients with CD4 counts of less than 200 cell/mm^3^ died of AIDS-defining conditions than those with CD4 counts of more than 200 cells/mm^3^ (75.86% versus 31.25%; *p* < 0.05). Furthermore, more patients who were on HAART in the study group died of a non-AIDS-defining illness than pre-HAART patients, and the relationship is statistically significant (75.00% versus 25.00%; *p* = 0.006).

**TABLE 4:** Association between gender and disease categorisation.

Variable	Non-AIDS	AIDS	Total	*p*-value
**Gender**				
Male	11 (34.38)	83 (57.24)	94	*p* = 0.019
Female	21 (65.63)	62 (42.76)	83	
Total	32	145	177	
**Age grouping**				
15–24	3 (9.38)	19 (13.10)	22	
25–44	15 (46.88)	74 (51.03)	89	
45–64	13 (40.63)	42 (28.97)	55	
65 +	1 (3.13)	10 (6.9)	11	
Total	32	145	177	*p* = 0.546
**CD4**				
< 200	10 (31.25)	110 (75.86)	120	
> 200	22 (68.75)	35 (24.14)	57	*p* < 0.01
Total	32	145	177	
**Duration of stay**				
< 1	39 (26.7)	9 (28.13)	48	
1–14	77 (53.10)	16 (50.0)	93	
15–28	17 (11.72)	4 (12.5)	21	*p* = 0.99
> 28 days	12 (8.28)	3 (9.38)	15	
Total	145	32	177	
**HAART status**				
HAART	24 (75.0)	70 (48.8)	94	*p* = 0.006
Pre-HAART	8 (25.0)	75 (51.1)	83	
Total	32	145	177	

## Discussion

This study demonstrated that the overall percentage of all deaths in the hospital’s medical wards directly attributable to HIV was relatively high at 35.0%, although this finding was consistent with studies in similar settings.^5,6,7^ As some patients died without an established HIV diagnosis, this percentage is also likely to be an underestimate.

Patient-related modifiable factors played a significant role through delayed presentation and defaulting on treatment, which led to patients’ presenting with more advanced disease that was more difficult to treat successfully.^6,7,17^ Other patient-related factors identified in the study were male gender, low CD4 counts of less than 200 cells/mm^3^ and pre-HAART patients, which could reflect determinants or predictors of HIV-related mortality. A similar trend has also been observed in other studies.^6,7,18^

In our setup, the focus of HIV and/or AIDS prevention strategies is largely on the pregnant woman (83.0% coverage) during antenatal care and the natal and postnatal periods, which in turn capture more HIV and/or AIDS-infected woman than men. The overall HAART coverage in Namibia is estimated at 88.0% coverage and the country has adapted the updated WHO eligibility criteria to CD4 500 cell/mm^3^.^3,4,5^ However, the mortality rate of HIV-related death is still high. The reasons for such late presentation and poor adherence identified in other studies include barriers to accessing care, forgetfulness, socio-economic constraints, fear of discrimination or stigmatisation, drug toxicity and limited knowledge of HIV and/or AIDS.^6,7,13,15,19,20^ As in our study, it is reported that early HIV diagnosis, early help-seeking behaviour, improved access to care, prompt initiation of HAART for eligible patients, and maintaining good adherence are critical to successful HIV and/or AIDS treatment.^7,19,21,17,22,23,24^ Further studies are needed to identify barriers to accessing care in our setting and to understand help-seeking behaviour in local communities.

Numerous researchers have reported significant reductions in mortality in HIV and/or AIDS patients after the introduction of HAART and attention having been given to modifiable factors in the quality of care.^6,8,9,13,14,15,16,25,18,26,27^

Modifiable factors identified in this study have highlighted several areas for potential improvement in adult care, treatment and monitoring. In our study, patients who had CD4 counts of more than 200 cells/mm^3^ and were on HAART died of non-AIDS-defining diseases, whereas the pre-HAART group succumbed due to AIDS-related diseases. This reflects the efficacy of HAART in preventing advanced HIV and/or AIDS disease; however, long-term use of HAART predisposes patients to metabolic complications which are also detrimental, a phenomenon being observed in developing countries.

Within the healthcare system at the hospital, there appeared to be discrimination against patients with HIV in terms of access to intensive care beds. Such discrimination might be related to a shortage of ICU beds, a lack of HIV and/or AIDS knowledge and negative attitudes towards critically ill patients. Similar findings on HIV and ICU care have been noted in some developing countries, whilst in South Africa, India and Brazil there are regulatory bodies that govern and prevent non-discriminatory practice.^28^ Therefore there is a need for policy-makers, health system administrators and clinicians to devise policy frameworks or guidelines to ensure non-discriminatory access to ICU care regardless of HIV status.

A lack of dialysis for HIV-positive patients with renal failure contributed significantly to deaths, as such patients depend mainly on conservative management. The importance of dialysis and close monitoring of renal function in patients with renal impairment cannot be over-emphasised, as there has been a rapid increase in renal failure in the HIV population worldwide. The HIV virus and Tenofovir-based therapy are both associated with renal diseases.^29^ There appears to be a lack of insight into and prioritisation of procurement for patients with HIV, although discussions with the management team revealed plans to construct a new dialysis unit by 2018. In the meantime, in-service training for peritoneal dialysis is being conducted as a short-term solution.

Health system managers must also ensure that essential consumables such as medication are available and should improve the turnaround time for crucial laboratory results. Blood products such as platelets take 48–72 hours to arrive from Windhoek and there is a need to construct a blood bank to serve northern Namibia.

Many patients who died were eligible for HAART, which reflects a gap in existing strategies for the screening, prevention and treatment of opportunistic infections at primary healthcare level. There is a need for our HAART centres at Oshakati and surrounding district hospitals to implement quality improvement projects that address this issue.^4,30^

The hospital relies on many foreign-trained healthcare workers who may not be familiar with HIV management. Therefore there is a need to familiarise junior colleagues with local guidelines and expose them to the HAART service with the objective of improving their HIV and/or AIDS case management skills.^30^ Deficiencies in individual clinical care may be addressed by more effective continuing professional development as well as regular risk management meetings that discuss morbidity and mortality. The hospital should make attendance at educational meetings mandatory and ensure more effective interactive small-group learning as opposed to formal didactic approaches. Disciplinary measures should be taken against healthcare workers who do not follow ethical rules and continue to practise negligently. Several studies have shown that greater physician experience and expertise is associated with reduced patient morbidity and mortality.^25,31^

Not all of the medical records could be retrieved and we also faced challenges arising from, information being missing from medical records. Data on the social, demographic and economic status of the patients was often not available in the folders and therefore the effect on health-seeking practice could not be studied. The clinical notes in some files were written in poor handwriting, which made data collection difficult. The causes of death were based on clinical findings recorded in the notes and no postmortems were conducted; this was mainly due to social–cultural barriers. The findings from postmortem examinations could have better defined the causes death.

Globally there are challenges in coding HIV deaths; added to which the available tools do not reflect the common conditions affecting sub-Saharan Africa. We overcame this difficulty in our study by using both the WHO and the CoDe to capture all causes.

## Conclusion

HIV and/or AIDS contributed significantly to overall inpatient mortality, and AIDS-defining diseases were the leading causes of deaths in the Oshakati Intermediate Hospital. The most common causes of death were extra-pulmonary tuberculosis, renal failure and *Pneumocystis jirovecii* pneumonia. Patient- and caregiver-related factors, particularly delays in seeking healthcare, were the most common modifiable factors. Access to critical care, dialysis and ventilator support as well as the poor monitoring of patients were the main modifiable factors in the hospital. Future studies should investigate issues related to the accessibility of healthcare and the initiation of HAART at primary care facilities. Continuing professional development, routine risk management, and better planning and procurement of essential resources will help to reduce deaths in the hospital.
